# Transcriptome profiling and environmental linkage to salinity across *Salicornia europaea* vegetation

**DOI:** 10.1186/s12870-019-2032-3

**Published:** 2019-10-16

**Authors:** Bliss Ursula Furtado, Istvan Nagy, Torben Asp, Jarosław Tyburski, Monika Skorupa, Marcin Gołębiewski, Piotr Hulisz, Katarzyna Hrynkiewicz

**Affiliations:** 1 Department of Microbiology, Faculty of Biology and Environmental Protection, Nicolaus Copernicus University, Lwowska 1, 87-100 Toruń, Poland; 2 Interdisciplinary Center for Modern Technologies, Nicolaus Copernicus University, Wileńska 4, 87-100 Toruń, Poland; 3 Department of Molecular Biology and Genetics, Science and Technology, Aarhus University, 4200 Slagelse, Denmark; 4 Chair of Plant Physiology and Biotechnology, Nicolaus Copernicus University, Lwowska 1, 87-100 Toruń, Poland; 5 Department of Soil Science and Landscape Management, Faculty of Earth Sciences and Spatial Management, Nicolaus Copernicus University, Lwowska 1, 87-100 Toruń, Poland

**Keywords:** Soil salinity, Halophyte, Glasswort, Next-generation sequencing, Season, Salt ions

## Abstract

**Background:**

*Salicornia europaea,* a succulent obligatory halophyte is the most salt-tolerant plant species in the world. It survives salt concentrations of more than 1 M. Therefore, it is a suitable model plant to identify genes involved in salt tolerance mechanisms that can be used for the improvement of crops. The changes in a plant’s gene expression in response to abiotic stresses may depend on factors like soil conditions at the site, seasonality, etc. To date, experiments were performed to study the gene expression of *S. europaea* only under controlled conditions. Conversely, the present study investigates the transcriptome and physicochemical parameters of *S. europaea* shoots and roots from two different types of saline ecosystems growing under natural conditions.

**Results:**

The level of soil salinity was higher at the naturally saline site than at the anthropogenic saline site. The parameters such as EC_e_, Na^+^, Cl^−^, Ca^+^, SO_4_^2−^ and HCO_3_^−^ of the soils and plant organs significantly varied according to sites and seasons. We found that Na^+^ mainly accumulated in shoots, whereas K^+^ and Ca^2+^ levels were higher in roots throughout the growing period. Moreover, changes in *S. europaea* gene expression were more prominent in seasons, than sites and plant organs. The 30 differentially expressed genes included enzymes for synthesis of S-adenosyl methionine, CP47 of light-harvesting complex II, photosystem I proteins, Hsp70 gene, ATP-dependent Clp proteases, ribulose bisphosphate carboxylase/oxygenase (Rubisco), phenylalanine ammonia-lyase (PAL), cytochrome c oxidase (COX) and ATP synthase.

**Conclusion:**

The comparisons made based on two seasons, plant organs and two different sites suggest the importance of seasonal variations in gene expression of *S. europaea*. We identify the genes that may play an important role in acclimation to season-dependent changes of salinity. The genes were involved in processes such as osmotic adjustment, energy metabolism and photosynthesis.

## Background

Soil salinity is one of the main environmental factors affecting the persistence of plants. Salinity stress in plants is generally considered as unique among abiotic stresses, in that it has two effector components: ionic imbalance and dehydration, which lead to multiple effects via osmotic stress, induced water deficits, ion toxicity, nutrient imbalance, etc. thus limiting plant growth and productivity [1]. In our study, we chose to characterize *Salicornia europaea* L. growing at two sites differing both in salinity level and salinization history (one naturally saline site with higher salinity level and second anthropogenically saline site with lower salinity level). The other soil physicochemical properties were similar. Detailed description of both sites was given in our previous paper [2]. We choose the sites because we wanted to see the differences in *S. europaea* transcriptome in different seasons (growth stages) due to different level and origin of salinity. In our earlier study we have seen seasonal differences in endophytic bacterial communities [2], which suggest concomitant changes in gene expression patterns. Therefore, this approach might reveal different salinity tolerance mechanisms operating at particular developmental stages (seedling and young plants vs. senescing ones).

In general, the only plants that show no evidence of growth inhibition in response to salinity or even exhibit salt requirement for optimal growth are the halophytes [3, 4]. Halophytic plants are common in coastal ecosystems around the world and represent diverse adaptations to hypersaline environments. It is a small group of plants well adapted to high salinity with species belonging mostly to Amaranthaceae, and a few to Plumbaginaceae, Plantaginaceae, Aizoaceae, Poaceae, and Brassicaceae [5]. The eHALOPH database (http://www.sussex.ac.uk/affiliates/halophytes/) currently identifies more than 1500 plant species reported from different parts of the world.

*S. europaea* (*herbacea*) L. (Amaranthaceae) (common names: glasswort, saltwort, marsh samphire) is one of the most salt-accumulating halophytes known as a “pioneer plant” found in coastal and inland saline sites [6]. They have specific morphological features that enable them to adapt to saline conditions [6, 7]. This genus comprises around 25–30 species that are widely distributed around the world [7]. This plant has generated significant interest as a multi-purpose plant which is of commercial value and ecological importance. It is suitable for cultivation in highly saline environments [8], as a source of secondary metabolites [9] and can be grown in aquaculture systems [10]. This halophyte can accumulate high amounts of Na^+^ (approx. 200 mM) in its shoots [11] compared to some salt-excreting halophytes [12]. Hence, this species is promising for desalination of salt-affected soils. Therefore, assessing the influence of salinity on *S. europaea* transcriptome may help us to understand mechanisms involved in soil desalinization and in this way increase the remediation efficiency [6–13]. *S. europaea* was also found to effectively accumulate inorganic nitrogen from wastewater [13] . Due to its salt tolerance, short generation time, its capability of producing many seeds and its high agronomic value, *Salicornia* is a valuable model species for exploring the salt tolerance mechanisms.

A number of adaptive traits of halophytes are expressed during the growing period that allows them to germinate, grow and complete their life cycle under high salt conditions [5]. The adverse effects of salinity influence almost all growth stages and physiological processes in plants, including photosynthesis [14], protein synthesis [15], energy production [16] and lipid metabolism [17]. Glycophytes, to which most of the known crop plants belong, are able to tolerate much lower salinity (maximum of 5 g dm^− 3^ of total dissolved solid (TDS), e.g. wheat (*Triticum aestivum*), Indian mustard (*Brassica juncea*) and barley (*Hordeum vulgare*) [18–20], while halophytes can tolerate levels as high as 1.3 M NaCl (twice the salinity of seawater), e.g. *Salicornia bigelovii* [21]. They employ different mechanisms to withstand salinity: (i) salt ions are compartmentalized, so that concentrations in the cytoplasm are maintained within tolerable limits, (ii) adjustment of their internal water relations through salt exclusion, (iii) succulence, (iv) salt-secreting glands and bladders, (v) selectivity of ion uptake and (vi) accumulation of compatible organic solutes [5, 22]. Salinity affects plants in multiple ways [23]. In addition, most of the mechanisms underlying salt stress are not universal and are dependent on the metabolic background and the biochemical pathways of the plant species [24].

The plant’s adaptive processes are mainly triggered in response to a changing environment which is initiated by their transcriptome, thus enabling the plant to attain cellular homeostasis through a series of molecular events [25]. Research on salt tolerance mechanisms of halophytes have been investigated through proteomic networks [26, 27] and transcriptome studies [28–30] which have reported several genes related to salt tolerance. Most of the available transcriptome data under salinity conditions are based on comparisons using model plant species [31]. Few reports are available on *S. europaea* genes: SePSY (phytoene synthase gene) [32], SeNHX1 (Vacuolar Na+/H+ antiporters) [33], SeLCY (beta-lycopene cyclase gene) [34] and SeCMO (choline monooxygenase) [35] that were introduced in model plants to demonstrate their gene expression under salt conditions. However, these data are limited to experiments conducted in model plants and not reported in *S. europaea* which is insufficient to accurately describe the molecular mechanisms involved in *Salicornia* salt tolerance. The lack of whole-genome sequence data presents difficulties in considering these plant species for use in understanding their response to salt stress at the molecular level and if studies have been carried out they are under controlled growth conditions [36, 37]. Thus, the goal of this study was to characterize transcriptome differences (i.e. to identify differentially expressed genes) stemming from differing salinity levels at the test sites. We hypothesized that the differences in the soil salinity, correlated with the seasonal variations, are the key drivers of gene expression response in *S. europaea*. To address this hypothesis, we sequenced the transcriptome of *S. europaea* roots and shoots coming from two test sites differing in salinity and analyzed the plant and soil physicochemical properties.

## Results

### Distribution of ions in soil and plant samples of *S. europaea*

Fall 2015 and spring 2016 clearly differed in terms of the selected meteorological parameters but were typical for the respective seasons in this area (Additional file 1 Table A). Higher soil salinity in the first period (fall 2015) compared to the second (spring 2016) can be explained by the generally lower rainfall sum and number of rainy days as well as higher mean air temperature. Comparisons of the soil parameters showed significant differences in EC_e_ and ion content which distinctly varied among the seasons at the sites (S1 and S2) (Table 2). The EC_e_ (electrical conductivity) of soil was higher at S1 with 100.5 dS m^− 1^ than at S2 (76.00 dS m^− 1^) during fall 2015 as compared to spring 2016. This trend can be explained by higher concentrations of such ions like Na^+^, Mg^+^, Cl^−^ and CaCO_3_ content at S1 during fall 2015.

The ion content in the *S. europaea* significantly differed between the shoot and root and between the two seasons at both sites (Table 3). A significant reduction in the amount of K^+^ ions with increasing accumulation of Na^+^ and Cl^−^ in the shoots was observed, whereas Ca^2+^ decreased and Mg^2+^ remained unchanged. The concentration of Cl^−^ was 2 times higher than Na^+^ in shoots for all samples. Concentration of Na^+^ and Cl^−^ ions in the roots was significantly increased during spring compared to fall. The Ca^+ 2^ and Cl^−^ content in roots reached higher values at S2 (103 mg g^− 1^ in fall and 166 mg g^− 1^ in spring), while at S1 it did not exceed 85 mg g^− 1^ (43 mg g^− 1^ in fall and 85 mg g^− 1^ in spring), respectively.

The concentration of ions present in the plant organs (shoots and roots) was positively related to the concentration of salts present in the soil, wherein the Na^+^, Cl^−^, K^+^, Ca^2+^ and Mg^2+^ in the plant organs were found to increase significantly with an increase in the soil ion concentrations. It can be estimated that the absorption of these ions by the plant reached approximately one-third of that found in soil. Overall, there were significant differences between sites (seven variables), but seasonality was even more important factor (ten variables).

### Quality assessment and de novo assembly of *S. europaea* RNASeq reads

The RNA from shoot samples of the S2 in spring was of poor quality; hence it was eliminated from the analysis. A total of 63 million and 45 million paired-end reads (75 nt) for the two data sets S1_FS and S2_FS (sites: S1 and S2; seasons: F (fall) and S (spring)) were generated with the Illumina MiSeq. Following trimming, the reads having ≥70% of the bases with a quality score ≥ Q20 were chosen for the downstream analyses. Reads from the two datasets were assembled separately and the assemblies contained a total of 181,809 and 180,401 transcripts with a N50 value of 1228 bp and 1360 bp for the S1_FS and S2_FS, respectively.

### Characterization of *S. europaea* S1_FS and S2_FS transcriptomes

**Fig. 1:**
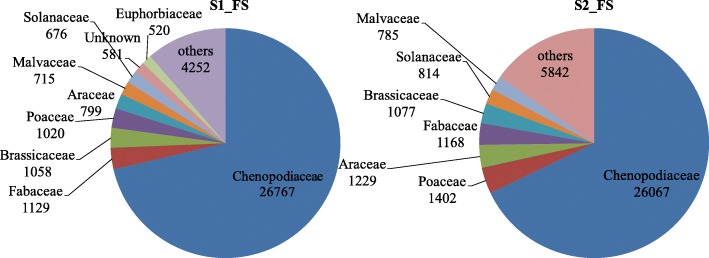
Classification of *S. europaea* assembled transcripts in the kingdom level classification obtained from NCBI Blastp database

**Fig. 2:**
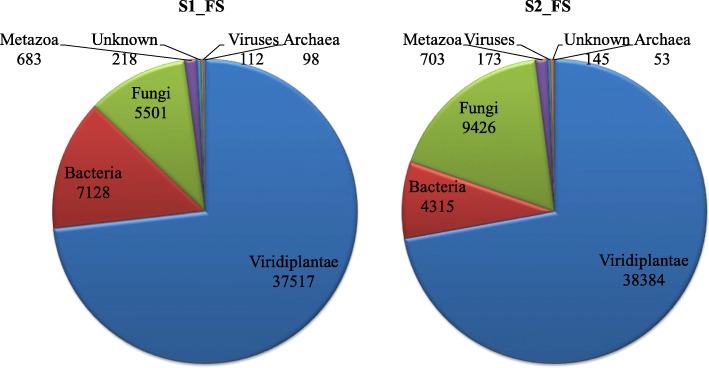
Top-hits of the family level distribution of *S. europaea* transcriptome

Comparing our de novo assembly to publicly available protein sequences showed a high portion of our assembled contigs to represent full-length coding sequences. Based on protein sequence similarity of protein-coding transcripts, a taxonomic classification was performed from the UniProt/SwissProt databases. Blastp hits were obtained for 51,257 contigs for dataset S1_FS and 53,199 contigs for dataset S2_FS. Among these, 37,517 (S1_FS) and 38,384 (S2_FS) sequences belonged to the Kingdom Viridiplantae. Classification at the plant family level showed the top of that the majority (more than 70%) of the *S. europaea* transcript assembly contigs were assigned to the family Chenopodiaceae (Fig. 2). The list of protein sequences of plant origin derived from *S. europaea* transcriptome (38,384 genes) with details on the closest match to other plant species and family are given in Additional file 4. Additionally, in the case of both investigated datasets, blastp analysis indicated a relatively high portion of transcript assembly contigs of sequences of non-plant origin, mainly coming from Bacteria and Fungi (Fig. 1).

**Fig. 3:**
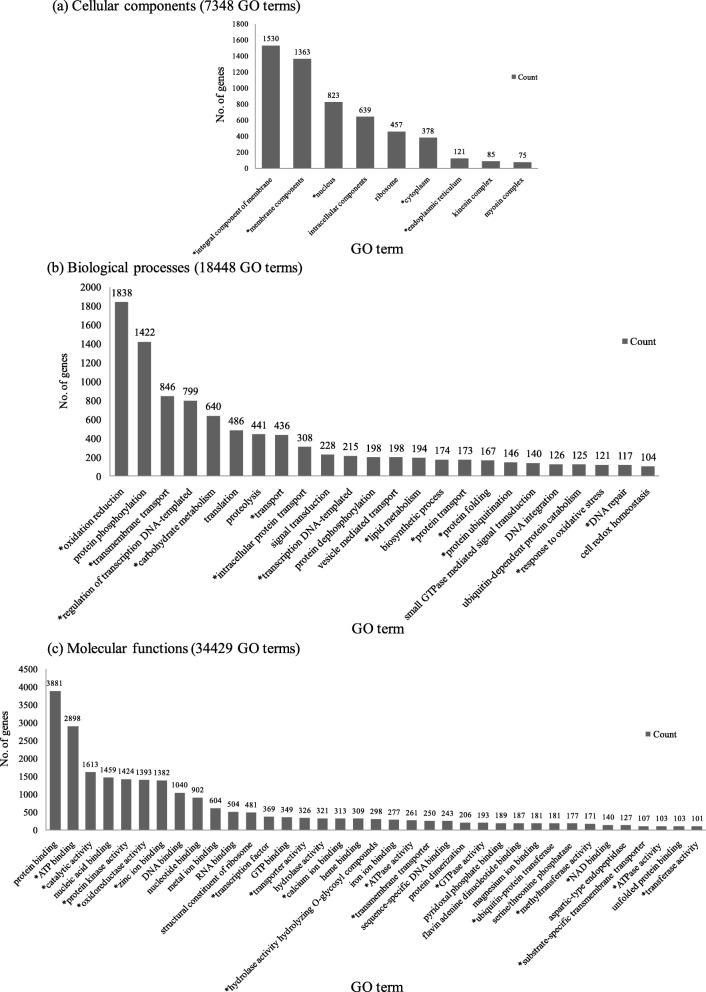
Functional classification of gene ontology terms in the total assembled *S. europaea* transcriptome. **a** cellular compartments (7348 GO terms), (**b**) biological processes (18,448 GO terms) and (**c**) molecular functions (34,429 GO terms) found in the total assembled *S. europaea* transcriptome. Note: GO terms less than 50 are given in the Additional file 3. GO terms that are known to contribute or express in response to salinity conditions are marked with an “*”

**Fig. 4:**
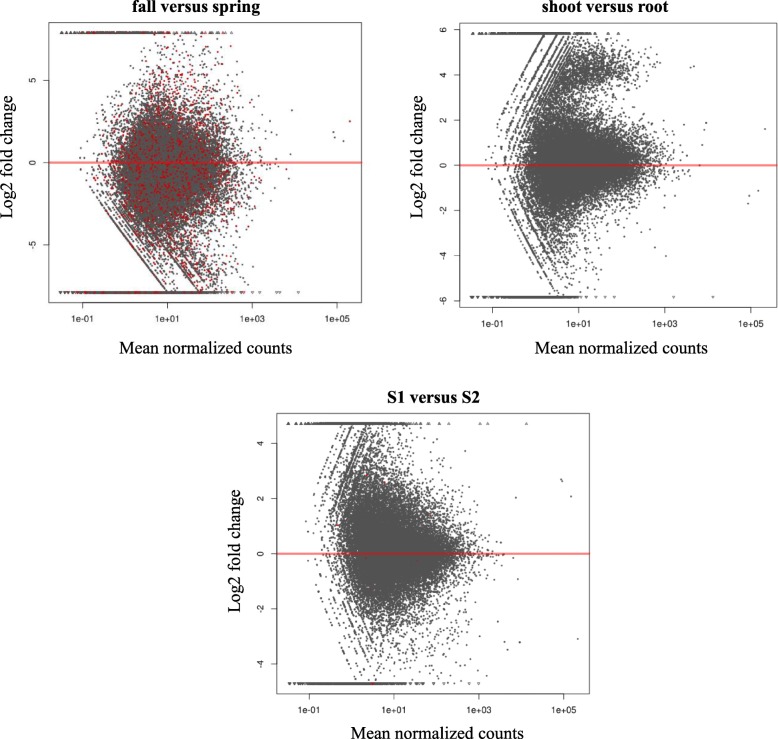
MA plot shows the log2 fold changes between two conditions for each factor (seasons, plant organs and sites). The log2 fold-change indicates the mean expression level for each gene (each dot represents a gene). The x axis is the normalized mean of all samples, the y axis the log2 fold change between the two conditions i.e. fall versus spring (seasons), shoot versus root (plant organs) and S1 versus S2 (sites). Red dots indicate genes with q value < 0.1

**Fig. 5:**
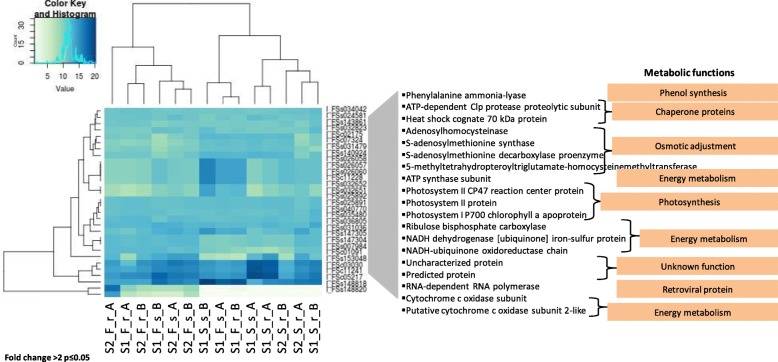
Hierarchically clustered heat map of 30 differentially expressed genes. The samples are clustered into 8 variants in two replicates: - site-(S1 and S2), plant organ- shoots (s) and roots (r) and season- fall (F) and spring (S). Potential functions were assigned to gene products via BLASTP against all UniProtKB/TrEMBL Viridiplantae protein sequences clustered with CD-HIT at 75% similarity level

### Functional annotation of protein-coding genes of *S. europaea*

Protein sequences of plant origin derived from the S2_FS reference transcriptome (38,384 sequences) were subjected to InterProScan analysis. Functional domain and protein family data obtained from InterProScan output along with the blastp top-hit descriptions were assigned to the sequences (Additional file 4). In total, 354,966 conserved domains and signatures could be identified on 36,409 protein sequences. Signal peptides and/or transmembrane domains could be found on 9148 sequences. Non-redundant Gene Ontology (GO) -term data were collected for each protein sequence from InterProScan outputs using custom scripts. A total of 60,225 GO-terms could be assigned to 23,180 protein sequences. Regarding the principal Gene Ontology domains, 34,429 GO-terms belonged to Molecular Function, 18,448 GO-terms to Biological Process and 7348 GO-terms to Cellular Compartments (Additional file 2). The total number of uniquely assigned GO terms was 221 for the cellular compartment, 735 for biological process and 894 for molecular function. Figure 3 shows the most represented GO terms from each of the three domains and GO terms with less than 50 genes are provided in the Additional file 2.

In the category of cellular components the greatest number of genes belonged to ‘integral component of cell membrane’ (1530 genes) and ‘membrane components’ (1363 genes). The biological process comprised ‘oxidation-reduction’ (1838 genes) and ‘protein phosphorylation’ processes (1422 genes) while the molecular function consisted mainly of protein binding (3881 genes) and ATP binding (2898 genes).

### Expression profiling and identification of differentially expressed genes (DEG)

The MA plot (Fig. 4) displays the log_2_ fold changes against the normalized mean of samples comparing gene expression for the two seasons: fall versus spring, plant organs: shoot versus root and two sites: S1 versus S2. The red dots mark genes detected as differentially expressed at 10% false discovery rate i.e. q value < 0.1 using Benjamini-Hochberg multiple testing *p*-value adjustment [38]. The points that fall out of the window are plotted as open triangles pointing either up or down in the top and bottom end of the graph. The dots above and below the zero (marked with red line) represent up and down-regulated genes. A high number of significant q-values were seen in the comparison of the two seasons, followed by location and plant organ.

Then, we applied the variance stabilizing transformation (VST) which performs a monotonous mapping such that for the transformed values, the variance is (approximately) independent of the mean. This approach was applied to create a sample clustering heatmap for all the samples. The heatmap demonstrated similar observations as the plant and soil data wherein the gene expression in *S. europaea* was significantly influenced by the seasonal variations when compared to the other 2 factors in this study. This analysis confirmed the significance of the seasonal variations in the gene expression of *S. europaea*.

A list of all the differentially expressed genes obtained in this study with the description of their GO terms, functional annotations and closest match to plant species are given in Additional file 4. The low number of DEG in our analysis could be explained by the variability among replicates or probably due to the lower gene counts which is not sufficient to yield significant up and down-regulated gene expression data for each of the factors in this study [39]. The top thirty differentially expressed genes (DEG) found in all samples (Fig. 5 and Additional file 3) (fold change > 2 at *p* ≤ 0.05) were classified into 8 main metabolic functions associated with the *S. europaea* salt response. Genes involved in transcription and posttranslational modifications i.e. RNA-dependent RNA polymerase, a gene for a Heat shock cognate 70 kDa protein were differentially expressed in all samples. An S-adenosylmethionine decarboxylase proenzyme along with some unknown proteins was over-expressed in the root (r) samples of S2 during spring (S) (S2_S_r_A and S2_S_r_B). The expression of ribulose bisphosphate carboxylase was higher in shoots (s) from S1 during spring (S) (S1_S_s_A and S1_S_s_B).

We further analyzed the DEGs for their GO annotations (Fig. 5) between each variant of the experiment. In all samples, 5-methyl tetrahydropteroyl triglutamate homocysteine methyltransferase, S-adenosyl homocysteinase, ATP synthase subunit, ATP-dependent Clp protease proteolytic subunit, cytochrome c oxidase subunit, heat shock cognate 70 kDa protein, NADH dehydrogenase [ubiquinone] iron-sulfur protein, particularly showed slight differences in expression. The S2_S_r had a high representation of genes that were recognized as “Uncharacterized protein” with unknown function.

The RT-qPCR analysis of five selected genes from the 30 DEGs demonstrated that the trends of gene expression from the RT-qPCR analysis were consistent with the RNA sequencing analysis (Additional file 5).

## Discussion

### Environmental data and physicochemical parameters of *S. europaea* were linked to the seasonal variations

The soil at S1 was classified as mineral (< 10% organic matter content), and at S2 was mineral-organic (10–20% organic matter content) [40]. As expected, soil salinity was significantly higher during fall, with low annual precipitation and higher air temperature, as compared to the spring; which may have resulted in higher evapo-transpiration and water deficit. However, the annual climatic conditions at the two sampling sites were quite similar during the two years of this study. The fall 2015 sampling was preceded by less rainfall, as compared to the spring 2016, which was very rainy resulting in higher humidity. Therefore, comparing all soil and meteorological data, EC_e_ was higher in fall 2015 and *S. europaea* was subjected to high salinity levels. Hence, we think that it is plausible to compare two different generations as the plants grew under similar conditions (see Supplementary materials). The differences in soil and plant samples physicochemical parameters (EC_e_ and ion levels) were more pronounced when seasons were compared than between sites. These results are consistent with the previous reports on the soil collected during fall 2013 at S2 which was dominated by Ca^2+^ ions (Ca^2+^ > Na^+^» Mg^2+^ > K^+^) and S1 with Na^+^ ions (Na^+^» Ca^2+^ > Mg^2+^ > K^+^) [41]. The pH of the soil at both sites was close to neutral, regardless of the season. A notable increase in organic matter content occurring during spring in the two salt-affected sites was likely caused by plant litter decomposition which was caused by the microbial population in soil (previously reported by [41]). Allochthonous supply of organic matter during high water levels also cannot be excluded [42].

Many studies have shown that the increasing concentrations of NaCl favors the growth and increases the water content in *S. europaea* [27, 36, 43]. In agreement with previous reports, our study confirms that *S. europaea* is an excellent salt accumulator and has substantial amounts of Na^+^ in its shoots making it an ideal candidate for phytoremediation [24, 44]. *Salicornia* sp. accumulates Na^+^ ions to accelerate water uptake when water is scarce, which maintains the osmolarity in cells [45]. *S. europaea* shoots are succulent, which allows for increased water uptake, and reduces ionic stress by maintaining the ion balance and cell integrity [46]. In order to maintain the osmotic gradient for water uptake from the soil, many halophytic plants roots accumulate organic ions to a concentration equal or greater to that in the soil [47]. Halophytes adaptation to saline environments largely depends on ion homeostasis that involves uptake, and distribution of mineral ions, toxic ion exclusion and sequestration of excess ions into vacuoles [11, 48]. In the present study, ion analysis revealed that Na^+^ and Cl^−^ more preferably accumulate in shoots, whereas Ca^2+^ and K^+^ ions are found in roots. Similar observations were made in other halophytes of the Chenopodiaceae family, where much higher content of Na^+^ than K^+^ was found, e.g. in *S. europaea* [11, 49], *Sueada maritima* [50], *Sueada aegyptiaca* [51], and *Atriplex micrantha* [52]. Our results show that the shoots of *S. europaea* effectively employs the ion uptake and compartmentalization mechanisms to selectively accumulate Na^+^ and Cl^−^ to the aerial parts of the plants, which is contrary to other halophytes, such as *Phragmites karka*, *Atriplex canescens*, and *Acacia auriculiformis* [53–55] that have active ion exclusion and translocation mechanisms to reduce Na^+^ ions in leaves.

### Quality and reliability of the de novo assembled transcriptome

To date, there was only a handful of reports describing the transcriptome of halophyte *S. europaea* that mainly focused on designing plants under controlled conditions and following salt treatment [27, 36, 37]. Ma et al. 2013 [36] provided insights into the molecular basis of salt adaptation in *S. europaea* shoots from controlled growth conditions. In contrast, our aim was to perform physicochemical analysis of soil and plant samples as well as transcriptome analysis of *S. europaea* shoots and roots collected directly from the natural environment. This facilitates construction of a reliable reference transcriptome makes it available for future analysis.

The robustness of the assembled *S. europaea* transcriptome was demonstrated by the high proportion of functional annotations of the transcript sequences and their high-level similarity to public database sequences. Numerous non-plant transcripts obtained in the de novo assembly (that often represent serious problems if field samples would be used for transcriptomic experiments) were effectively excluded from further analyses by taxonomic analysis of transcript sequences. For this purpose, we classified the transcripts based on their taxonomy and using the Gene Ontology (GO) analysis that describes the gene function at the cellular, biological and molecular level (http://geneontology.org).

#### Seasonal variations influence *S. europaea* gene expression under salinity

Many pathways involved in plant responses to salt stress may be conserved, but their relative importance may vary with species, varieties, and even tissues [23, 56]. The differences in the concentration of ions (Na^+^, Cl^−^, K^+^, Ca^+^, Mg^+^, SO_4_^2−^ and HCO_3_^−^) in soil and plants were consistent according to seasons at the two investigated sites. Likewise, differences in the gene expression profiles among the two seasons were initially observed through the MA plot with genes significantly expressed among the two seasons, and further analyzed by sample clustering heatmap. The genes hierarchically clustered according to their gene expression values and the samples grouped according to the seasons (fall and spring), irrespective of site and plant organ. Genes I_FSs148818 and I_FSs148820 (Additional file 4) with sequence name: RNA-dependent RNA polymerase, showed very low gene expression (*p* values < 5) in the plant samples in spring compared to samples in fall. Notably, the genes I_FSc05217 (Photosystem II protein), I_FSc03030 (Predicted protein), I_FSs153048 (Uncharacterized protein) and I_FSc11241 (Uncharacterized protein) showed p values ranging from 13 to 20 in all samples in spring while the gene expression with p value < 10 in fall samples. The observed changes in photosynthetic pigment proteins (PS-II) in *S. europaea* could have been induced by the shortening of the daily light period during fall as well as increasing salt accumulation in the plant might have decreased photosynthetic processes [57]. A similar observation was made by Tiku and colleagues in halophyte *Salicornia rubra* and *Distichlis stricta* photosynthesis and biomass production under different light intensities and osmotic conditions where increasing NaCl concentration decreased the chlorophyll concentration of Salicornia and increased that of Distichlis [58]. The reason for the down-regulation of RNA-dependent RNA polymerase and up-regulation of uncharacterized proteins needs further investigation. However, we obtained a small number of DEGs probably due to a high degree of environmental variation or because of the low read depths per sample obtained during sequencing, which indicates that short read data obtained by the MiSeq sequencing technology are not optimal for quantitative expression profiling.

We selected the 30 DEGs found in all comparisons. Among these we found six DEGs (designated as uncharacterized proteins) with no assigned functional annotation. They require further investigation. The other 24 genes might be involved in salt tolerance of *S. europaea,* as they were engaged in osmotic adjustment, ion compartmentalization, photosynthetic adaptation and in accumulation of osmolytes [48]. Based on this background we classified the DEGs into 5 categories according to their metabolic functions. The first category consisted of DEGs involved in osmotic adjustment, the enzyme 5-methyl tetrahydropteroyl triglutamate-homocysteine methyltransferase was previously reported to be salt responsive at the mRNA level [59]. This enzyme is responsible for the regeneration of S-adenosyl methionine (SAM) by plants under salt stress [60] and is an important methylating agent involved in flowering and lignin biosynthesis [59]. The phenomenon of increased lignification was detected in water- or salt-stressed plants [61] including *Salicornia* [27]. SAM is also involved in betaine synthesis which is an osmoprotectant and is reported to play a role in ion homeostasis in *S. europaea* [27]. The second category of DEGs represents photosynthetic machinery: chlorophyll a and b binding proteins (CP47) of the light-harvesting complex II and photosystem I proteins. The photosynthetic electron transport chain is the main source of reactive oxygen species (ROS) in plants that can damage the photosynthetic machinery via stress-induced leakage of electrons to oxygen (O_2_) [62]. A significant induction in photosynthetic genes, PSI and PSII pigment binding proteins, b6f complex and ATPase synthase CF1 was reported in salt treated plants of *S. europaea* [37]. Third category of DEGs comprised the Hsp70 gene involved in thermotolerance that was previously reported by Augustine et al. to increase sugarcane resistance to salinity and drought when overexpressed [63]. The ATP-dependent Clp protease proteolytic subunit also falls in this category. It is a protease involved in plant defense from stress, having a prominent role in dis-aggregation of the protein upon increasing temperatures [64, 65]. The fourth category of genes belonged to energy metabolism that was the Ribulosebisphosphate carboxylase/oxygenase (RubisCO) enzyme complex [66] that plays an important role in photosynthetic acclimation to moderate heat stress in vivo. An enhanced degradation of RubisCO subunits has been observed in several glycophytic plants and crops exposed to salt stress [65, 67]. The regulation of cytochrome c oxidase (COX) under stress conditions may be important in energy generation through the respiratory chain. An increased relative abundance of cytochrome c oxidase subunit 6b-1 was observed in the roots of salt-treated rice [68]. Plants response to salinity poses enhanced demands on energy production, resulting in an increase of ATP synthase subunits namely, subunit β that have been detected in several salt-treated plants [65, 69, 70]. NADH dehydrogenasees are involved in the mechanism of response to nitro- oxidative stress [71]. Sobhanian et al. reported the downregulation of NADH dehydrogenase 1 beta subcomplex subunit 8 in soybean seedlings exposed to salt stress suggested a decrease in the ATP pool which resulted in decreased plant growth [67]. Lastly, a DEG involved in defense metabolism- phenylalanine ammonia-lyase (PAL) was detected in this study. It is a key enzyme in pathogen defense, stress response and secondary metabolism [72]. A strong and positive correlation between PAL and phenolic compounds was observed at different salt concentrations in treated roots of *Morinda citrifolia* [73].

Remediation of salt-affected soils is not always cost-effective, therefore many researchers and farmers are shifting towards saline agriculture, which stresses the importance of studies on halophytic crops one [37, 48]. Similarly, data on the mechanisms by which halophytes survive and maintain productivity can be useful to develop tolerant varieties in conventional crops [33, 45]. Understanding the salinity tolerance mechanisms operating in halophytes, with respect to their environmental conditions and growth stages could maximize the use of halophytes capacity to accumulate and exclude salts in an effective way (50, 51).

## Conclusions

The reference transcriptome generated in this study can be a useful tool for other researchers as, judging from its high full-length coding sequences content, it is of high quality. The physicochemical and transcriptomic analyses emphasize the role of the seasonality and salinity, correlated with the former, in shaping *S. europaea* transcriptome. Three basic cellular processes were found to be affected by seasonality in our study, two of which (energy metabolism and photosynthesis) were probably related to seasonality per se, while the third one (osmotic adjustment) to salinity, which in turn was season-dependent.

## Method

### Sample collection

The plant samples of halophyte *S. europaea* were collected from two salt affected areas in two seasons (fall 2015 and spring 2016). The salt-affected areas are located in Central Poland (Table 1): site 1 (S1) is located in the vicinity of three brine concentration towers in the Spa Park in the town of Ciechocinek (natural source of salinity) and site 2 (S2) is a meadow next to waste ponds of a soda factory in Inowroclaw (anthropogenic source of salinity).

**Table 1:** Details on the two selected sampling locations in Central Poland

Site	Location	Type of the salinity source	Geographical coordinates	Cause of salinity
S1	Ciechocinek	natural	N52°53, E18°47°	The periodic flooding by brine (NaCl) transported by a ditch from the graduation towers (previously taken from the natural spring being in contact with Zechstein rock-salt deposits).
S2	Inowrocław	anthropogenic	N52°48, E18°15°	Salt infiltration (mainly CaCl_2_ and NaCl) from the improperly sealed waste ponds. There are solid and semi-liquid solid industrial waste produced during the manufacturing of the soda ash by the Solvay method.

**Table 2:** Chemical parameters of soil collected from two salt-affected sites during fall 2015 and spring 2016

	Site 1		Site 2	
	fall 2015	spring 2016	fall 2015	spring 2016
ECe	100.5 ± 27.6^b**#**^	51.1 ± 12.7^a^	76.0 ± 19.5^b^	59.7 ± 12.2^a^
pH_e_	6.8 ± 0.1^a^	7.8 ± 0.1^a^	6.9 ± 0.1^a^	7.3 ± 0.1^a^
Na^+^ [g∙dm^−3^]	21.5 ± 7.9^a**#**^	9.2 ± 2.4^b#^	11.8 ± 7.4^b^	7.4 ± 2.1^b^
Cl^−^ [g∙dm^−3^]	65.3 ± 21.6^a#^	30.8 ± 5.9^b^	44.1 ± 13.4^b^	34.2 ± 5.6^b^
Ca^2+^ [g∙dm^−3^]	4.2 ± 3.5^b^	0.9 ± 0.2^a^	8.1 ± 3.3^a#^	7.6 ± 1.5^a#^
K^+^ [g∙dm^−3^]	0.4 ± 0.2^a^	0.2 ± 0.0^c^	0.2 ± 0.2^b^	0.2 ± 0.1^b^
Mg^2+^ [g∙dm^−3^]	0.5 ± 0.2^a^	0.2 ± 0.1^b^	0.3 ± 0.2^b^	0.0 ± 0.0^a^
SO_4_^2−^ [g∙dm^−3^]	0.3 ± 0.085^a^	0.8 ± 0.2^b^	0.1 ± 0.1^a^	0.6 ± 0.3^b^
HCO_3_^−^ [g∙dm^−3^]	0.1 ± 0.0^a^	0.2 ± 0.1^a^	0.1 ± 0.0^a^	0.1 ± 0.0^a^
SP [%]	94.5 ± 14.1^a#^	83.0 ± 9.3^b^	89.4 ± 10.5^a^	133.1 ± 48.6^b#^
TOC [%]	5.9 ± 2.5^a^	4.8 ± 3.1^a^	7.5 ± 5.5^b#^	3.3 ± 2.4^a^
carbonates [%]	39.4 ± 7.1^a#^	33.9 ± 9.4^a#^	28.4 ± 10.5^b^	23.1 ± 2.0^a^

Values are a mean ± standard error (*n* = 9). Values labeled with letters show significant differences between two seasons in the same site. The same letters show no significant difference (*p* ≤ 0.05). Values with “#” depict higher and significant difference between two sites in the same season. Abbreviations: EC_e_- electrical conductivity; TOC-total organic carbon; SP- saturation percent

**Table 3:** Ion concentrations of *S. europaea* shoots and roots from the two salt-affected sites in different seasons

	Site 1				Site 2			
	fall 2015		spring 2016		fall 2015		spring 2016	
mg.g^−1^	Shoot	Root	Shoot	Root	Shoot	Root	Shoot	Root
Na^+^	No sample	2.0 ± 1.2	149 ± 25.2^a^	50.5 ± 6.6^b#^	81.4 ± 8.7^a#^	4.6 ± 2.8^b^	60.3 ± 5.9^a^	34.5 ± 9.2^b#^
K^+^		1.3 ± 0.8	18.2 ± 0.8^a^	12.4 ± 0.2^b#^	14.4 ± 2.0^a^	7.2 ± 6.0^b^	14.8 ± 1.9^a^	21.3 ± 5.0^b#^
Cl^−^		48.2 ± 27.5	240.3 ± 38.5^a^	85.2 ± 8.5^b#^	145.4 ± 15.0^a^	123.7 ± 30.2^b#^	166 ± 14.0^a#^	65.8 ± 13.5^b^
Ca^+^		43.3 ± 27.4^#^	10.4 ± 0.6^b^	6.6 ± 1.3^b^	59.0 ± 11.0^a^	103.0 ± 29.0^b#^	44.3 ± 1.3^a^	9.3 ± 0.8^b^
Mg^+^		9.1 ± 1.0^#^	2.65 ± 0.1^b^	1.1 ± 0.1^b^	2.4 ± 0.4^a^	13.8 ± 0.3^b#^	0.43 ± 0.1^b^	0.5 ± 0.0^b^

Values are a mean ± standard error (n = 9). Values labeled with “^a^” and “^b^” show significant differences between shoot and root. The same letters are not significantly different (p ≤ 0.05). Values marked with “#” show significant differences between organs from the two sites (p ≤ 0.05)

According to the Köppen-Geiger classification the study sites, like most of Polish territory, are located in temperate, fully humid with warm summer [74]. The data from the meteorological station in Toruń (2015–2016; www.imgw.pl), located closest to both study sites (30–40 km), was used for the climatic characteristics (Additional file 1 Table A). The average annual temperature for both years was 9.9 and 9.5 °C, respectively, and the annual rainfall was 379.4 and 680.2 mm. Our plant samples come from wild and protected area and we obtained permission from Regional Directorate for Environmental Protection in Bydgoszcz. The formal identification of the samples was done by K. Hrynkiewicz. No voucher specimens were collected and deposited in the collection (it is not necessary as we don’t describe a novel species). Field studies were conducted in accordance with local and EU legislation.

Three plots (10 × 10 m, biological replicates) were chosen at random at each site S1 and S2. Three blocks of soil (20 × 20 × 20 cm) along with *S. europaea* were randomly sampled from each plot in each season: fall [F] and spring [S]. For molecular analysis, 1 g of plant samples (shoots and roots) were washed with sterile distilled water to separate soil debris, immediately frozen in liquid nitrogen and stored at − 80 °C until further processing. At the same time, the soil and plant (shoots and roots) samples were analyzed for physicochemical data.

### Quantification of ion content in plant shoots and roots and soil samples

Soil samples (3 samples/plot) collected from three plots (*n* = 9) in each test site during the two seasons. The soil samples were air-dried and passed through a 2 mm mesh sieve to remove large debris. The soil was analyzed for the following: total organic carbon (TOC) (CNS Variomax analyzer), and carbonates (Scheibler volumetric method) [75]. The saturation paste extracts were prepared to evaluate the electrical conductivity (EC_e_) (conductometric method), pH_e_ (potentiometric method) and saturation percentage (SP) (gravimetrically) [76]. Moreover, the ion content in the extracts were determined: calcium (Ca^2+^), magnesium (Mg^2+^), sodium (Na^+^), potassium (K^+^), and chloride (Cl^−^) by as described by van Reeuwijk (2002) [76].

Plant samples (3 samples/plot) collected from three plots (*n* = 9) in each test site during the two seasons. The plant samples (shoots and roots) were prepared for chemical analysis in the following: homogenization, drying at 60 °C, hot mineralization using the mixture of HNO_3_ and H_2_O_2_and dry combustion at 460 °C [77, 78]. The total content of Na^+^, K^+^, Ca^2+^, Cl^−^ and Mg^2+^was determined with the methods described for the soil extracts. Statistical analysis (ANOVA) for the soil, shoot and root data was performed by Statistica version 7. Data for shoot samples collected from S1 during fall 2015 are not provided in the results.

### Total RNA extraction

Total RNA was extracted from 500 mg of frozen plant tissue from the roots (r) and shoots (s) using TriPure reagent (Roche) followed by DNase (DNase I,Thermo Scientific). The quantity and quality of RNA was checked using Qubit RNA HS assay kit and Qubit fluorometer (Life Technologies) and the RNA 6000 NanoAssay Kit and Agilent 2100 Bioanalyzer (Agilent Technologies). The total RNA extract was stored at − 70 °C for later use.

Based on bioanalyzer results (RIN values > 8) we selected 42 samples for sequencing. We did not obtain a high yield and good quality of RNA for shoot samples from the S2 during spring and hence these samples were not analyzed.

### cDNA library preparation and sequencing

Libraries were prepared from 1 μg of total RNA using TruSeq stranded total RNA sample preparation kits (Illumina), according to the producer’s instructions. Following synthesis, libraries were subjected to quantification and quality control by means of Bioanalyzer DNA HS assay and by qPCR using KAPPA Illumina library quantification kit (Kappa Biosystems). Finally, the libraries (6 libraries per run) were sequenced on the Illumina MiSeq instrument.

### De novo assembly

The initial quality check of the raw sequence data was performed using FastQC v.0.11.3 (http://www.bioinformatics.babraham.ac.uk/projects/fastqc/). Adapter sequences and low-quality terminal nucleotides were removed using Trimmomatic (v0.33) [79]. Read pairs longer than 60 nt were kept for further processing. In order to create reference transcript assemblies, trimmed short reads were assembled separately for the two sampling sites using the de novo assembler Trinity (v2.3.2) [80] resulting in two assembled data sets “S1_FS” and “S2_FS” (F is for fall and S is for spring samples respectively).

Further, we analyzed the gene expression between *Salicornia* populations at S1 and S2 habitats. The S2_FS dataset was chosen as a common reference for profiling since the N50 value of the S2_FS transcripts was calculated to be 1360 bp, which was higher than the N50 value of the S1_FS transcripts (1228 bp). Therefore, the S2_FS transcript assembly is likely to represent a higher amount of full-length transcripts. Only plant-specific, protein-coding transcripts (transcript with best BLAST hits coming from Viridiplantae) were included in the reference dataset, in order to minimize false discovery rates that might be strongly influenced by environmental transcript contamination.

### Functional annotation and gene ontology assignment

Protein coding transcript sequences were predicted using GeneMark S-T [81]. Predicted protein sequences were subjected to *blastp* searches against reference protein sequences. Blast searches were performed on a local server. An E-value cutoff of 1e-3 was applied and top hit sequences were collected for further comparative analyses. For target reference sequences a reduced redundancy plant protein sequences database (all UniProtKB/TrEMBL Viridiplantae protein sequences clustered at 75% similarity level, resulted in 4,098,635 sequences) plus 5, 53,231 sequences from UniProt/SwissProt were used. Taxonomy information was assigned to UniProt top hits using custom scripts.

Protein sequences were further analyzed using the InterPro databases for their protein family relationships, signal peptides and transmembrane domains, and Gene Ontology (GO) terms via local searches (InterProScan-5.16-55.0, [82]). Per gene, GO-term information was collected from the InterProScan outputs using custom scripts.

### Quantitative expression profiling

To increase the read depth per sample for DESeq analysis [39], samples were pooled to produce two biological replicates: A and B for all 8 variants of the experiment (two test sites: S1 and S2, two seasons: fall (F) and spring (S), two organs: shoots (s) and roots (r); 16 variants in total). The shoot samples of S2 during spring were eliminated for sequencing (because of low quality RNA), hence 14 variants were analyzed finally. Quantitative gene-level expression profiling of plant-specific transcript assembly contigs from the S2_FS reference assembly was performed by Kallisto [83] by applying 100 bootstrapping steps. Normalized read count data obtained by Kallisto were further processed by Sleuth [84] and DESeq [39]. Differentially expressed genes were determined by factorial comparisons of samples from the S1 and S2 locations through Variance Stabilizing Transformation.

### Validation of DEG by quantitative RT-PCR (RT-qPCR)

RT-qPCR was performed to quantify the expression levels of *selected DEG found in this study*. A total of 1 μg of total RNA was used in cDNA synthesis according to the manufacturer’s instructions (Transcriptor HighFidelity cDNA Synthesis Kit (Roche)). The RT-qPCR reaction was performed on LightCycler® 480 using FastStart SYBR Green I Master following the manufacturer’s protocol (Roche). The primers were selected from the 30 differentially expressed genes in this study and designed using Primer3 [85] (Additional file 1 Table B). The polyubiquitin gene was used as a reference [86] (Additional file 1 Table B). Three independent biological and three technical replicates were analyzed. The relative fold expression was calculated using the Pfaffl method [87].

## Supplementary information

**Additional file 1: Table A.** Meteorological data of the two salt affected sites. Average monthly temperature (t), relative humidity (RH), precipitation (p) and number of rainy days (NRD) in 2015–2016 (meteorological station in Toruń). Data source: Institute of Meteorology and Water Management – National Research Institute (IMGW-PIB). Table B Primer sequences for RT-qPCR validation of *S. europaea* genes. Table C Meteorological data of the two salt affected sites. The table below presents the long-term average from the period 1981–2010 (meteorological station in Toruń). Data source: Institute of Meteorology and Water Management – National Research Institute (IMGW-PIB) http://www.pogodynka.pl/polska/daneklimatyczne/.

**Additional file 2. **GO terms classification for the total sequences of *S. europaea* transcriptome. (a) cellular component (7348 GO terms), (b) biological processes (18,448 GO terms) and (c) molecular function (34,429 GO terms).

**Additional file 3. **Summary of selected 30 differentially expressed genes (DEGs) in *S. europaea* with their molecular function (MF) and biological processes (BP).

**Additional file 4. **List of differentially expressed genes (DEG) obtained in this study. The gene list [protein sequences of plant origin derived transcriptome (38,384 genes)] along with the description of their GO terms, functional annotations and closest match to plant species are given. The TPM (Transcript Per kilobase Million) values are the mean ± stdev for pooled replicates A and B (*n* = 2) for each variant of the experiment.

**Additional file 5.** RT-qPCR analyses for validation of RNA sequencing data. Bar graphs with the relative expression ratio against sample variants is plotted for RT-qPCR and RNA sequencing (RNAseq) values. Five genes from among the 30 differentially expressed genes were selected: - Cytochrome c oxidase subunit (CYT b), ATP synthase subunit (ATP), NADH-ubiquinone oxidoreductase chain (NADH), Ribulosebisphosphate carboxylase (RuBisCO) and Heat shock cognate 70 kDa protein (HSP).

## Data Availability

The raw RNA-Seq datasets generated during the current study are available in the NCBI repository Bioproject no. PRJNA436955. Biosample no. SAMN08687491 to SAMN08687532 via SRA accession no. SRP134955.

## Abbreviations

COX: Cytochrome c oxidase
DEG: Differentially expressed gene
EC_e_: Electrical conductivity
F: Fall
GO: Gene Ontology
MA plot: Mean and average scales plot
PAL: Phenylalanine ammonia-lyase
PS-II: Photosystem II
r: Root
ROS: Reactive oxygen species
RT-qPCR: Quantitative reverse transcription polymerase chain reaction
Rubisco: Ribulosebisphosphate carboxylase/oxygenase
s: Shoot
S: Spring
S1: Site 1
S2: Site 2
SAM: S-adenosyl methionine
TDS: Total dissolved solids
TOC: Total organic carbon
VST: Variance stabilizing transformation
