# Supplementary material for: Transcriptome profiling and environmental linkage to salinity across Salicornia europaea vegetation
Source: BMC Plant Biol. 2019 Oct 16;19:427. doi: 10.1186/s12870-019-2032-3 (PMC6794796; doi:10.1186/s12870-019-2032-3)
Supplement: Supplementary file 1 — Additional file 1: Table A. Meteorological data of the two salt affected sites. Average monthly temperature (t), relative humidity (RH), precipitation (p) and number of rainy days (NRD) in 2015–2016 (meteorological station in Toruń). Data source: Institute of Meteorology and Water Management – National Research Institute (IMGW-PIB). Table B Primer sequences for RT-qPCR validation of S. europaea genes. Table C Meteorological data of the two salt affected sites. The table below presents the long-term average from the period 1981–2010 (meteorological station in Toruń). Data source: Institute of Meteorology and Water Management – National Research Institute (IMGW-PIB) http://www.pogodynka.pl/polska/daneklimatyczne/. [file 12870_2019_2032_MOESM1_ESM.docx]

**Transcriptome profiling and environmental linkage to salinity across *Salicornia europaea* vegetation**

Bliss Ursula Furtado^1, 4^, Istvan Nagy^2^, Torben Asp^2^, Jarosław Tyburski^3, 4^, Monika Skorupa^4^, Marcin Gołębiewski^3, 4^, Piotr Hulisz^5^, Katarzyna Hrynkiewicz^1, 4*^

^1^Department of Microbiology, Faculty of Biology and Environmental Protection, Nicolaus Copernicus University, Lwowska 1, 87-100 Toruń, Poland

^2^Department of Molecular Biology and Genetics, Science and Technology, Aarhus University, 4200 Slagelse, Denmark

^3^Department of Plant Physiology and Biotechnology, Nicolaus Copernicus University, Lwowska 1, 87-100 Toruń, Poland

^4^Interdisciplinary Center for Modern Technologies, Nicolaus Copernicus University, Wileńska4, 87-100 Toruń, Poland

^5^Department of Soil Science and Landscape Management, Faculty of Earth Sciences, Nicolaus Copernicus University, Lwowska 1, 87-100 Toruń, Poland

Emails: Bliss Ursula Furtado [bliss.furtado@umk.pl](mailto:bliss.furtado@umk.pl), Istvan Nagy [istvan.nagy@mbg.au.dk](mailto:istvan.nagy@mbg.au.dk), Torben Asp [torben.asp@mbg.au.dk](mailto:torben.asp@mbg.au.dk), Jarosław Tyburski [tybr@umk.pl](mailto:tybr@umk.pl), Monika Skorupa [monika_skorupa@umk.pl](mailto:monika_skorupa@umk.pl), Marcin Gołębiewski [mgoleb@umk.pl](mailto:mgoleb@umk.pl), Piotr Hulisz [hulisz@umk.pl](mailto:hulisz@umk.pl), Katarzyna Hrynkiewicz* [hrynk@umk.pl](mailto:hrynk@umk.pl) *corresponding author

Additional file 1 Table A. Meteorological data of the two salt affected sites. Average monthly temperature (t). relative humidity (RH). precipitation (p) and number of rainy days (NRD) in 2015-2016 (meteorological station in Toruń). Data source: Institute of Meteorology and Water Management – National Research Institute (IMGW-PIB)

| 2015 | | | | | | | | | | | | | |
| --- | --- | --- | --- | --- | --- | --- | --- | --- | --- | --- | --- | --- | --- |
|  | I | II | III | IV | V | VI | VII | VIII | IX | X | XI | XII | year |
| t [°C] | 1.5 | 0.9 | 5.1 | 8.1 | 13 | 16.3 | 19.1 | 22.1 | 14.3 | 7.3 | 5.8 | 4.8 | 9.9 |
| RH [%] | 85.3 | 83.3 | 72.7 | 67 | 63.3 | 64 | 64.5 | 55.1 | 77.5 | 81.8 | 89.7 | 87.5 | 74.3 |
| p [mm] | 26.7 | 7.3 | 29.2 | 27.3 | 22.8 | 34.2 | 98.5 | 3.9 | 42.6 | 32.5 | 32.1 | 22.3 | 379.4 |
| NRD | 14 | 6 | 10 | 8 | 11 | 15 | 14 | 3 | 9 | 9 | 15 | 15 | 129 |
| 2016 | | | | | | | | | | | | | |
|  | I | II | III | IV | V | VI | VII | VIII | IX | X | XI | XII | year |
| t [°C] | -2.6 | 3.3 | 4.0 | 9.1 | 15.8 | 18.9 | 19.2 | 17.8 | 15.7 | 7.6 | 3 | 1.6 | 9.5 |
| RH [%] | 87.4 | 83.4 | 81.4 | 64.6 | 63.9 | 61.5 | 71.5 | 72.6 | 71.4 | 87.9 | 90 | 91.1 | 77.2 |
| p [mm] | 21.8 | 29.2 | 17.4 | 36.5 | 35.5 | 51.5 | 199.7 | 39.3 | 13.6 | 123.5 | 46.1 | 66.1 | 680.2 |
| NRD | 19 | 15 | 15 | 12 | 10 | 11 | 16 | 15 | 4 | 22 | 12 | 14 | 165 |

Additional file 1 Table B. Primer sequences for qRT-PCR validation of *S. europaea* genes.

| **Nr.** | **DEG_ID** | ***S. europaea* gene** | **primer sequence** | **Length** | **Tm** | **GC%** | **Product**  **size** | **Target_Seq**  **length** | **Start_on**  **Target** |
| --- | --- | --- | --- | --- | --- | --- | --- | --- | --- |
| 1 | I_FSs031479_F | Heat shock cognate 70 kDa protein | TGACAAGAAGGCAACCAGTG | 20 | 59.9 | 50 | 150 | 2514 | 777 |
|  | I_FSs031479_R |  | AAGTCCTCACCACCCAAATG | 20 | 59.8 | 50 |  |  | 926 |
| 2 | I_FSs025891_F | Cytochrome c oxidase subunit | GTGCTACGCCTTTTGGTAGC | 20 | 59.9 | 55 | 149 | 3650 | 662 |
|  | I_FSs025891_R |  | TGCATTGCCTTTTATGACCA | 20 | 60.1 | 40 |  |  | 810 |
| 3 | I_FSs034042_F | ATP synthase subunit | CTACCGGCATTTTACGGATG | 20 | 60.3 | 50 | 150 | 2320 | 853 |
|  | I_FSs034042_R |  | TAGAATCCATTTCGGGAACG | 20 | 59.9 | 45 |  |  | 1002 |
| 4 | I_FSs036805_F | NADH-ubiquinone oxidoreductase chain | CGCACCTCTGTATCGACTGA | 20 | 60 | 55 | 150 | 2908 | 688 |
|  | I_FSs036805_R |  | CTTGCCTCTTTGTGGGTGAT | 20 | 60.1 | 50 |  |  | 837 |
| 5 | I_FSc11228_F | Ribulosebisphosphate carboxylase | GAAAAGGGCGGATACGAAAT | 20 | 60.3 | 45 | 149 | 18994 | 15946 |
|  | I_FSc11228_R |  | CCCCCTATGTTGGGGTTACT | 20 | 59.9 | 55 |  |  | 16094 |
|  |  |  |  |  |  |  |  |  |  |
|  | Reference gene | Polyubiquitin | ACGCTGGAGGTCGAAACATC | 20 | 70.5 | 55 | 139 |  |  |
|  |  |  | TATAATCCGCCAGTGTCCTGC | 21 | 70.2 | 52.4 |  |  |  |

Additional file 1 Table C. Meteorological data of the two salt affected sites. The table below presents the long-term average from the period 1981-2010 (meteorological station in Toruń). Data source: Institute of Meteorology and Water Management – National Research Institute (IMGW-PIB) <http://www.pogodynka.pl/polska/daneklimatyczne/>

|  | **Absolute temperature (°C)** | | **Average monthly precipitation totals (mm)** | **The highest and lowest monthly rain sums (mm)** | |
| --- | --- | --- | --- | --- | --- |
|  | max | min |  | max | min |
| January | 12.8 | -32 | 29.8 | 82.5 | 1.7 |
| February | 17.1 | -28.7 | 26.3 | 66.5 | 4.9 |
| March | 21.5 | -22.3 | 31 | 51.1 | 5.7 |
| April | 28.9 | -8.3 | 30.1 | 103.4 | 0.5 |
| May | 32.3 | -6.2 | 50.7 | 119.8 | 12.3 |
| June | 35.5 | 0.7 | 57.8 | 115.6 | 19.3 |
| July | 37.9 | 3.1 | 84.2 | 226.8 | 12.8 |
| August | 37.5 | 3.7 | 67.3 | 159.2 | 2.7 |
| September | 31.1 | -2.8 | 51.5 | 155.4 | 7.3 |
| October | 25.6 | -10.1 | 33.3 | 110.1 | 4.3 |
| November | 17.4 | -20.1 | 36.9 | 124.8 | 10.5 |
| December | 13.2 | -22.9 | 38.5 | 92.9 | 6.6 |
