# Supplementary figures and images for: Transcriptome profiling and environmental linkage to salinity across Salicornia europaea vegetation
Source: BMC Plant Biol. 2019 Oct 16;19:427. doi: 10.1186/s12870-019-2032-3 (PMC6794796; doi:10.1186/s12870-019-2032-3)

## RT-qPCR

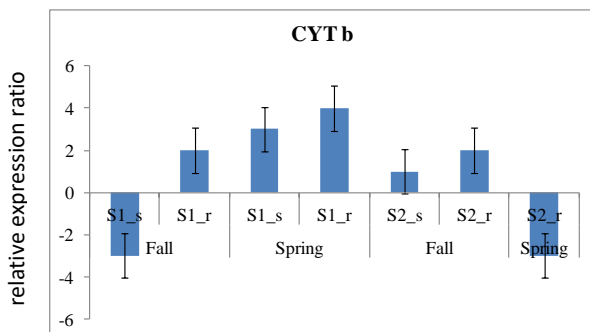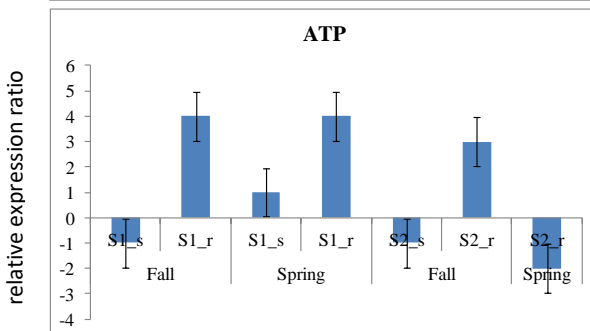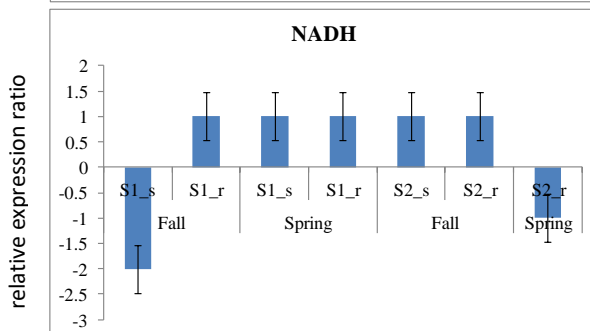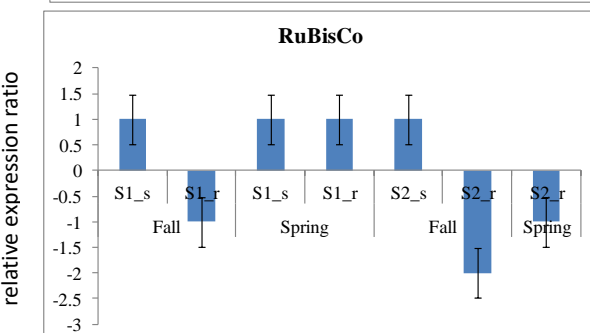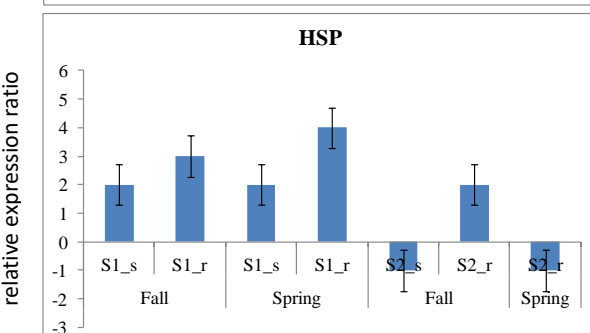

## RNAseq

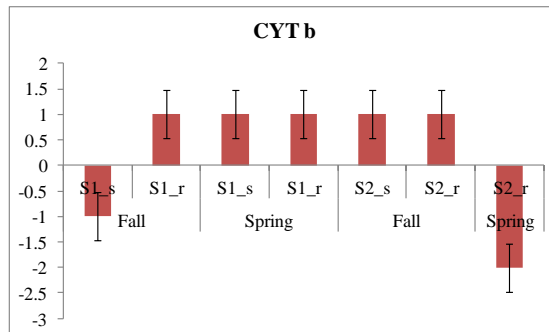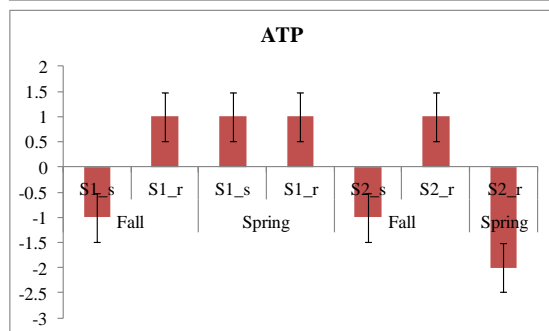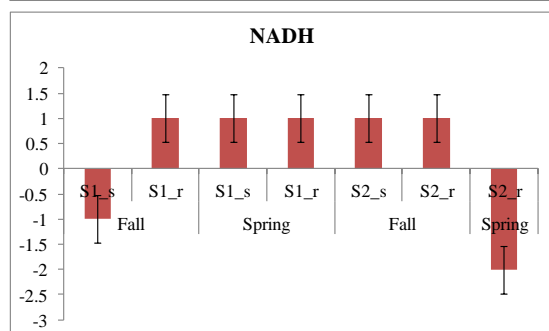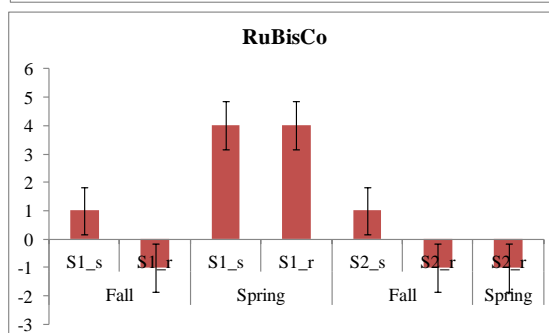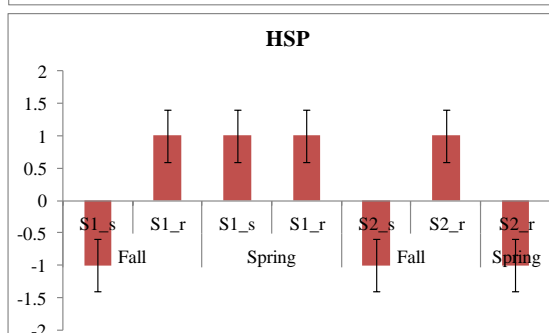

Supplement: Supplementary file 5 — Additional file 5. RT-qPCR analyses for validation of RNA sequencing data. Bar graphs with the relative expression ratio against sample variants is plotted for RT-qPCR and RNA sequencing (RNAseq) values. Five genes from among the 30 differentially expressed genes were selected: - Cytochrome c oxidase subunit (CYT b), ATP synthase subunit (ATP), NADH-ubiquinone oxidoreductase chain (NADH), Ribulosebisphosphate carboxylase (RuBisCO) and Heat shock cognate 70 kDa protein (HSP). [file 12870_2019_2032_MOESM5_ESM.pdf]
